# Elevation of Serum Spermidine in Obese Patients: Results from a Cross-Sectional and Follow-Up Study

**DOI:** 10.3390/nu14132613

**Published:** 2022-06-24

**Authors:** Hanshu Gao, Qianlong Zhang, Jiahui Xu, Wei Yuan, Ruixue Li, Hui Guo, Cuiying Gu, Wenjing Feng, Yanan Ma, Zhaoqing Sun, Liqiang Zheng

**Affiliations:** 1 Department of Biostatistics and Epidemiology, School of Public Health, China Medical University, Shenyang 110122, China; gaohanshu97@163.com (H.G.); 18241777233@163.com (W.Y.); liruixue2020@163.com (R.L.); guohui0471@126.com (H.G.); 2020120220@stu.cmu.edu.cn (C.G.); wenjing.feng@me.com (W.F.); ynma@cmu.edu.cn (Y.M.); 2 School of Public Health, Shanghai Jiao Tong University School of Medicine, Shanghai 200025, China; xujiahui1116@163.com; 3 Ministry of Education—Shanghai Key Laboratory of Children’s Environmental Health, Xinhua Hospital, Shanghai Jiao Tong University School of Medicine, Shanghai 200092, China; zhangql7989@163.com; 4 Institute of Health Sciences, China Medical University, Shenyang 110122, China; 5 Department of Cardiology, Shengjing Hospital of China Medical University, Shenyang 110004, China

**Keywords:** spermidine, obesity, overweight, compensatory, change in BMI

## Abstract

Background: Spermidine, a natural polyamine, appears to be a promising intervention for the treatment of obesity in animal studies, but epidemiological studies on the association between spermidine and obesity are inadequate. Methods: In the cross-sectional study, a total of 4230 eligible Chinese rural participants aged ≥ 35 years at baseline were recruited, of whom 1738 completed the two-year follow-up. Serum spermidines were measured using high-performance liquid chromatography with a fluorescence detector. Obesity and change in BMI were used as outcomes. Multivariable logistic regression analysis was applied to obtain the odds ratios (ORs) and 95% confidence intervals (CIs). Results: Participants who were obese had higher serum spermidine concentrations than those who were of normal weight (median (IQR), 27.2 ng/mL (14.8–53.4 ng/mL) vs. 23.8 ng/mL (12.8–46.6 ng/mL), *p* = 0.002). Compared with participants in the first quartile, those in the third quartile (OR 1.327, 95% CI 1.050 to 1.678) and the fourth quartile (OR 1.417, 95% CI 1.121 to 1.791) had a significantly increased risk of prevalent obesity after adjustment for confounding factors. In the follow-up study, participants in the third quartile (OR 0.712, 95% CI 0.535 to 0.946) and the fourth quartile (OR 0.493, 95% CI 0.370 to 0.657) had significantly lower risks of an increase in BMI after adjustment for confounding factors, with the lowest quartile as the reference. Meanwhile, we found a nonlinear relationship between spermidine and BMI in the follow-up study (*p* < 0.001). Conclusions: Serum spermidine was positively associated with increased odds of obesity in the cross-sectional study but reduced odds of an increase in BMI in the follow-up study among Chinese adults. Future studies are warranted to determine the exact mechanism underlying the association between spermidine and obesity and the scope for interventions.

## 1. Introduction

The obesity epidemic confronts the world as a major challenge, with an extensive impact on individuals and societies at large. A worldwide study showed that the prevalence of obesity doubled from 1980 to 2015 in more than 70 countries and continued to increase in most other countries [1]. In China, the prevalence of obesity increased from 3.1% in 2004 to 8.1% in 2018 [2]. Furthermore, obesity is an important risk factor for many chronic non-communicable diseases. Many studies have shown that obesity is strongly associated with a substantial increase in many major chronic diseases, such as cardiovascular diseases [3,4], diabetes [4,5,6], many types of cancer [7,8] and even all-cause mortality [9]. Although lifestyle interventions are considered as an effective treatment for obesity, they are difficult to maintain in terms of efficacy and continuity of treatment, especially in individuals who are severely obese or have multiple comorbidities. Therefore, the discovery of pharmacological interventions that mimic physiological enhancement is a research priority in the field of obesity.

Spermidine is the most abundant polyamine in the majority of different human tissues and it is essential for the maintenance of normal metabolic functions [10,11]. Spermidine in tissues can be synthesized intracellularly, ingested through the diet, or produced by the intestinal flora [12]. Spermidine is mainly found in some specific foods, such as wheat germ, soybeans and mushrooms [13]. Increased dietary intake of spermidine has been found to be associated with reduced all-cause mortality and the incidence of cardiovascular disease, while whole-blood spermidine levels decrease with aging, suggesting that spermidine may have health-protective effects associated with disease and aging [14,15,16]. While the current studies are insufficient to support spermidine as a treatment for diseases, spermidine still has a tremendous potential effect for adjunctive intervention.

Previous animal studies suggest that spermidine supplementation can help reduce body weight and improve metabolic disorders associated with obesity. In 2018, two animal studies consistently showed that spermidine supplementation caused a significant loss of weight and improved insulin resistance in high-fat diet-induced obese mice [17,18]. Furthermore, spermidine also can reduce the blood lipid level in high-fat diet-induced obese mice or acute hyperlipidemia mice [19,20,21,22]. In summary, animal studies have shown that spermidine supplementation appears to be a promising intervention for improving obesity. However, few studies have focused on spermidine and obesity in the human population. A large-scale cross-sectional population study showed that the daily intake of spermidine was negatively associated with body mass index (BMI) [17]. However, another study showed that obese patients with metabolic syndrome had elevated serum spermidine levels after bariatric surgery [23]. The effect of spermidine on obesity is an area of research requiring more investigation because the small body of epidemiological evidence to date is inconsistent.

In previous studies, the primary method of measuring spermidine concentrations in the body was through self-reported dietary scales, which may be subject to recall bias. Since spermidine in the body does not only originate from food, spermidine concentrations measured from human biological samples are more objective and reasonable. Therefore, to expand the limited epidemiological evidence in the field and provide evidence for future intervention experiments based on data from rural areas in China, we aimed to determine the effect of serum spermidine levels on obesity in general Chinese adults through a cross-sectional and follow-up study.

## 2. Materials and Methods

### 2.1. Study Population

Data were derived from a longitudinal cohort conducted in rural areas of Fuxin Autonomous County, Liaoning Province, China. We recruited participants from the eastern, southern, and northern townships to take part in our survey. A questionnaire survey of the general population was conducted from June to August 2019. Participants were eligible if: (1) they were 35 years of age or older; (2) they had stayed in the study area for at least five years; (3) they were willing to sign a consent form. After excluding participants based on the following reasons: (1) pregnant; (2) developing severe liver and renal failure; (3) unwilling to participate in this study, 4689 participants were recruited as the study population. The follow-up survey was conducted between March to June 2021. Finally, 2088 participants completed the follow-up survey. Data on demographics and other factors were recorded through interviews. Informed consent was obtained from all participants. If the participants were unable to write, their guardians would read and sign the informed consent form on their behalf. This study was approved by the human experimentation committee of China medical university.

**Figure 1:**
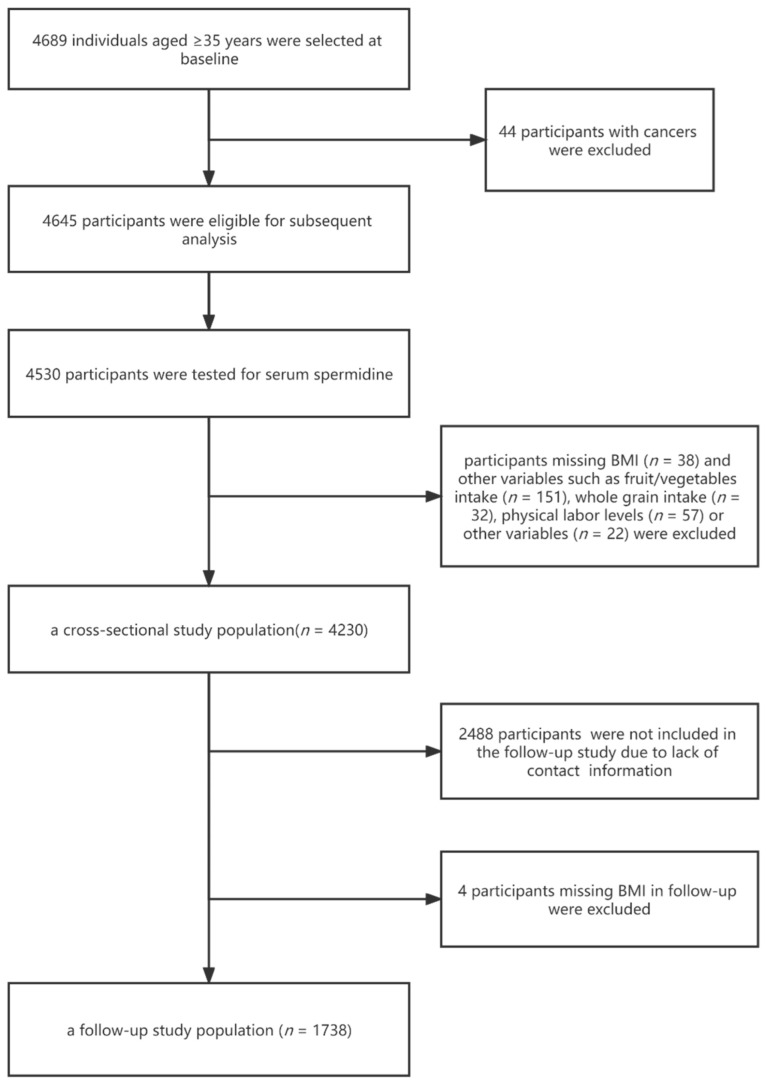
The study population inclusion and exclusion process.

For the present study, Figure 1 shows the participants’ specific inclusion and exclusion process. Of the 4689 participants at baseline, 44 participants with cancers were excluded. We measured serum spermidine concentrations in 4530 participants for subsequent analysis. Those without information about BMI (*n* = 38) and other variables, such as fruit/vegetables intake (*n* = 151), whole grain intake (*n* = 32), physical labor levels (*n* = 57) or other variables (*n* = 22) were excluded. The remaining 4230 participants were used for the cross-sectional study analysis. For the follow-up study, 2488 participants were not included due to a lack of contact information. Four participants were excluded at follow-up due to missing BMI. Finally, 1738 participants were included in the follow-up study analysis.

### 2.2. Detection of Spermidine in Serum Using High-Performance Liquid Chromatography with Fluorescence Detection (HPLC-FLD)

Participants were examined after an overnight fast. Blood samples were drawn from the antecubital vein in the morning and were collected in siliconized vacuum glass tubes. Serum was obtained by centrifugation for 10 min at 3000 rpm, 10,000× *g* and then stored at −80 ℃ (Department of Biobank, Shengjing Hospital of China Medical University) until analysis. Spermidine levels in the serum were measured using HPLC-FLD. Briefly, the main process of detection of serum spermidine was as follows: 200 μL 0.1 M HCL was added to 100 μL serum for the spermidine trihydrochloride assay. Then, protein precipitation was performed by adding 1 mL acetonitrile (ACN) to precipitate the serum protein. The extracted samples were analyzed using an HPLC system to obtain a standard curve after derivatization. The LC column was an Agilent TC-C18 column (250 mm × 4.6 mm, 5 µm particle size). Mobile phase solutions A and B were ultrapure water and ACN, respectively. Gradient elution was selected as follows: 0–7 min, 55–50% A; 7–25 min, 50–10% A; 25–31 min, 10% A; 31–35 min 10–55% A; 35–40 min 55% A. The flow rate was 0.8 mL/min. The column temperature was 35 ℃, and the detection wavelength was λex/λem = 340/510 nm.

### 2.3. Definition of Obesity and Overweight

In the cross-sectional and follow-up study, weight and height were measured and body mass index was calculated by trained researchers following standard operating procedures (RGZ-120). BMI (kg/m^2^) was calculated as body weight/(height^2^). Overweight and obesity were defined as having a BMI of 24–28 kg/m^2^ and ≥28 kg/m^2^. Overweight/obesity was defined as having any overweight or obesity (BMI ≥ 24 kg/m^2^). In the follow-up study, the change in BMI over two years was calculated as follow-up BMI minus baseline BMI.

### 2.4. Assessment of Other Variables

Data on demographic characteristics (age, sex, and ethnicity), lifestyle factors (smoking status, alcohol intake, and physical activity), and history of disease (such as hypertension, diabetes, stroke, and so on) were collected through face-to-face interviews with standardized questionnaires in the cross-sectional and follow-up study. The history of diseases was defined as self-reported and confirmed by medical records. Smokers were defined as people who smoked at least one cigarette per day and continued for at least half a year. The ingestion of alcohol was defined as at least three drinks every week for six months. Physical labor levels were divided into three levels: low, medium and high, depending on the occupation engaged in. Dietary intake of vegetables or fruits and whole grains was divided into four levels according to the frequency of intake.

Fasting blood samples were collected in the morning from participants who had fasted for at least 8 h. All laboratory equipment was calibrated, and the blood samples of all populations were randomly coded and tested blindly to effectively reduce systematic error and variability of laboratory batch measurements. Fasting plasma glucose and blood lipids were analyzed using the Roche Cobas 8000 C701 automatic biochemical analyzer in a central laboratory, and those methods are operated under the scope of accreditation. Diabetes mellitus was defined as fasting plasma glucose ≥ 7.0 mmol/L (≥126 mg/dL) or using hypoglycemic drugs or insulin [24].

We defined multimorbidity as the presence of at least two chronic conditions out of a predefined list of seven relevant chronic diseases [25]. The chronic diseases considered were coronary heart disease, stroke, diabetes, hypertension, chronic obstructive pulmonary disease, chronic hepatitis and liver cirrhosis.

### 2.5. Statistical Analysis

The continuous variables were taken as the mean ± standard deviation (SD) or the median (interquartile range) and compared using the one-way ANOVA test or Kruskal–Wallis H test. The categorical variables for independent proportions were expressed as the ratio of frequency and compared using Pearson’s χ^2^-tests.

Multivariate logistic regression models were used to estimate the odds ratios (ORs) and their 95% confidence intervals (CIs) for serum spermidine and overweight/obesity in the cross-sectional study. Serum spermidine was divided into quartiles (Q_1_: <13.58 ng/mL, Q_2_: 13.58–24.88 ng/mL, Q_3_: 24.88–49.65 ng/mL, Q_4_: ≥49.65 ng/mL) with the first quartile (Q1) as a reference. Models were computed for two separate weight status metrics: (i) obesity vs. not obesity; (ii) overweight/obesity vs. normal. Three different models were introduced: Model 1 without adjustment; Model 2, adjusted for gender and age and Model 3, additionally adjusted for gender, age, smoking, drinking, ethnicities, physical labor levels, fruit/vegetable intake levels, whole grain intake levels, triglyceride, total cholesterol, high density lipoprotein cholesterol (HDL-C), low density lipoprotein cholesterol (LDL-C), history of diabetes, and history of stroke. The associations between serum spermidine and BMI were evaluated on a continuous scale with restricted cubic spline curves based on linear regression models. To explore whether the relationship between serum spermidine and obesity was masked by obesity-related multimorbidity, we repeated the above-mentioned multivariate logistic regression in a subgroup analysis based on whether the participants were with multimorbidity.

In the follow-up study, multivariate logistic regression models were used to estimate ORs for baseline serum spermidine and the increase in BMI over two years. Serum spermidine was divided into quartiles (Q1: <13.18 ng/mL, Q2: 13.18–24.32 ng/mL, Q3: 24.32–48.56 ng/mL, Q4: ≥48.56 ng/mL) with the first quartile (Q1) as a reference. The three models additionally adjusted for baseline BMI on top of the above models. The associations between serum spermidine and the change in BMI over two years were evaluated on a continuous scale with restricted cubic spline curves based on linear regression models. As age, gender and weight status in the baseline survey are important influences for the increase in BMI, we performed subgroup analyses with study subjects stratified by age (<65 or ≥65 years), sex (male or female), and weight status in the baseline survey (normal, overweight or obesity), respectively.

All statistical calculations were performed using IBM SPSS statistical software version 23.0 (SPSS Inc., Chicago, IL, USA) and R 4.1.1. A two-sided *p*-value of <0.05 was considered statistically significant.

## 3. Results

The basic characteristics of the participants are described in Table 1, according to the weight status in the cross-sectional analyses. Of the 4230 participants included (mean age 59.2 ± 9.9 years, 64.2% females), 1652 were overweight, and 743 (17.6%) were obese. Compared with participants of normal weight, those who were overweight and obese had a higher prevalence of diabetes and stroke and showed higher triglyceride, total cholesterol, LDL-C and lower HDL-C (all *p* < 0.05). Moreover, participants who were overweight and obese had higher serum spermidine levels than those of normal weight (obesity vs. normal, *p* = 0.002).

**Figure 2:**
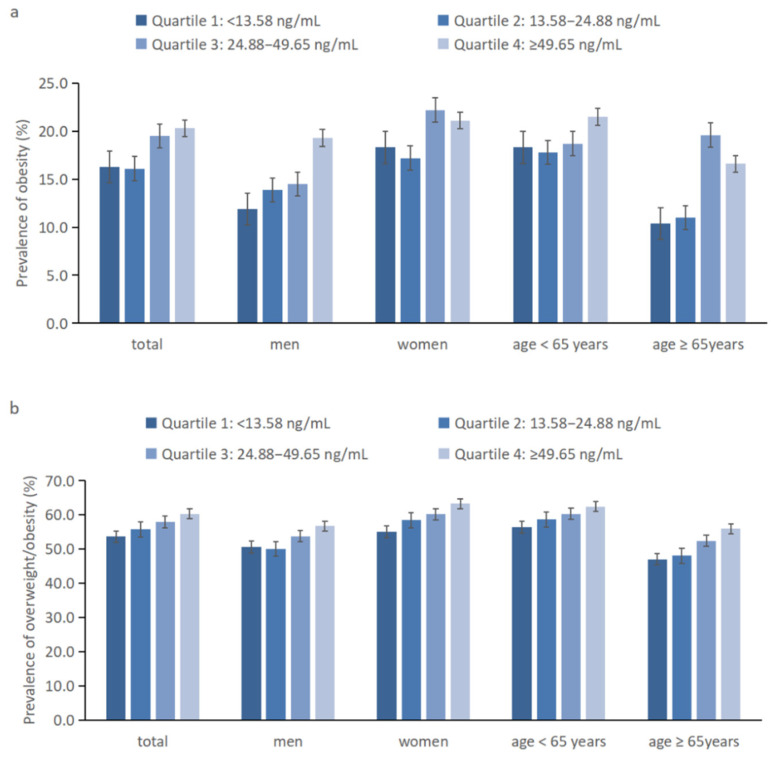
(**a**) Prevalence of obesity (BMI ≥ 28 kg/m^2^) at different serum spermidine levels in the general population and subgroups by sex and age. (**b**) Prevalence of overweight/obesity (BMI ≥ 24 kg/m^2^) at different serum spermidine levels in the general population and subgroups by sex and age.

The prevalence of obesity (BMI ≥ 28 kg/m^2^) and overweight/obesity (BMI ≥ 24 kg/m^2^) at different serum spermidine levels in the general population and subgroups by sex and age is shown in Figure 2. In the general population, for subjects in the first, second, third, and fourth quartiles of serum spermidine, the prevalence of obesity was 16.3%, 16.1%, 19.5%, and 20.3%, respectively. Similarly, for subjects in the first, second, third, and fourth quartiles of serum spermidine, the prevalence of overweight/obesity was 53.6%, 55.7%, 57.9%, 60.3%, respectively. Overall, the prevalence of obesity was higher in participants with high levels of spermidine and showed similar results in different subgroups of the population.

### 3.1. Association between Serum Spermidine and Obesity as Well as BMI in a Cross-Sectional Study

The association between serum spermidine and the prevalence of obesity and overweight/obesity is presented in Table 2. As shown, after assessing serum spermidine in quartiles, participants in the third quartile (OR 1.327, 95% CI 1.050 to 1.678) and the fourth quartile (OR 1.417, 95% CI 1.121 to 1.791) had significantly higher risks of obesity after adjustment for confounding factors than those in the first quartile. Furthermore, participants in the third quartile (OR 1.315, 95% CI 1.092 to 1.582) and the fourth quartile (OR 1.449, 95% CI 1.201 to 1.748) had significantly higher risks of overweight/obesity after adjustment for confounding factors than those in the first quartile.

To explore whether the relationship between serum spermidine and obesity was masked by obesity-related multimorbidity, a subgroup analysis is displayed in Supplementary Material Table S1. The results suggest that the above-mentioned association between spermidine and obesity or overweight/obesity is only seen in patients with obesity-related multimorbidity.

**Figure 3:**
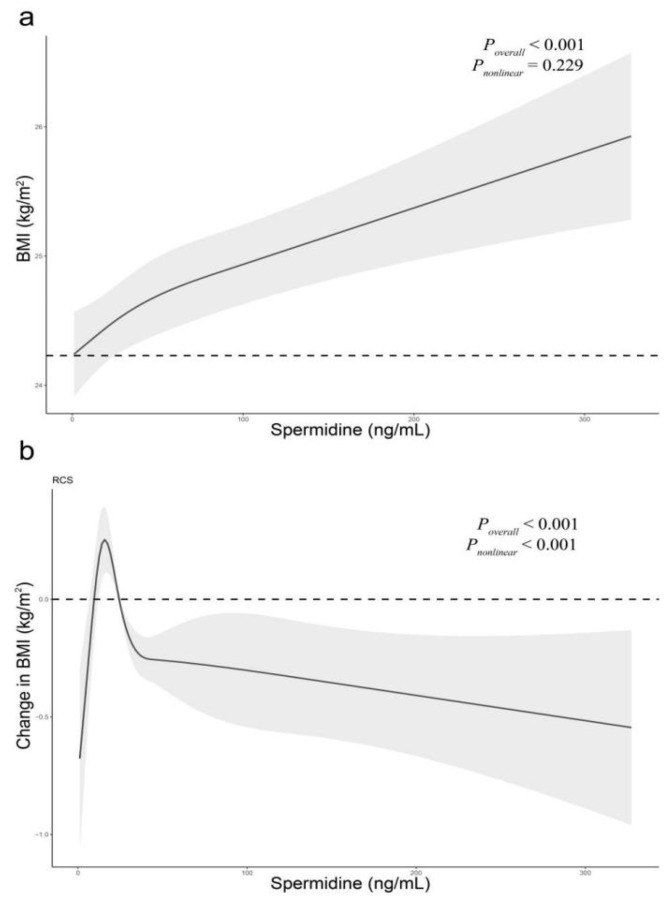
The relations of serum spermidine with the baseline BMI and change in BMI. In the cross-sectional study, we adjusted for gender, age, smoking, drinking, ethnicity, physical labor levels, fruit/vegetables intake levels, whole grain intake levels, triglyceride, HDL-C, LDL-C, total cholesterol, history of diabetes, and history of stroke. In the follow-up study, we additionally adjusted for baseline BMI compared to the cross-sectional study. (**a**), baseline BMI; (**b**), change in BMI.

Restricted cubic spline showed a dose-response relationship of serum spermidine (as a continuous variable) with BMI (*p* = 0.278 for the nonlinear test, Figure 3a). The result demonstrated a weak J-shaped association between serum spermidine and BMI, after adjusting for confounding factors.

### 3.2. Association of Serum Spermidine with the Change in BMI during a Follow-Up Study

As shown in Table 3, after assessing baseline serum spermidine in quartiles, participants in the third quartile (OR 0.712, 95% CI 0.535 to 0.946) and the fourth quartile (OR 0.493, 95% CI 0.370 to 0.657) had significantly lower risks of the increase in BMI after adjustment for confounding factors when compared with those in the first quartile. Furthermore, subgroup analyses among the associations are displayed in Supplementary Material Table S2. There were no modification effects of sex and age on the relationship between serum spermidine and the increase in BMI (all *p* ≥ 0.05). Notably, the relationships with the increase in BMI were more pronounced in overweight people.

Restricted cubic spline showed a dose-response relationship between baseline serum spermidine (as a continuous variable) and the change in BMI (*p* < 0.001 for the nonlinear test, Figure 3b). After adjusting for confounding factors, the result significantly demonstrated a reverse J-shaped association between the baseline serum spermidine and the change in BMI, with the peak point being 15.77 ng/mL.

## 4. Discussion

In Chinese rural adults, our study showed that (i) in the cross-sectional study, participants with higher serum spermidine concentration were at higher risk of prevalent obesity, and serum spermidine levels had a significant J-shaped association with BMI; (ii) moreover, on the basis of a 2-year follow-up, participants with higher baseline serum spermidine concentrations were more likely to decrease BMI than those with lower serum spermidine concentrations. This association was more pronounced in those who were overweight at baseline. Baseline serum spermidine levels had a significant reverse J-shaped association with the change in BMI.

Spermidine, as a natural polyamine that can be obtained from specific foods, has been found to play a key role in cell proliferation and is essential for the growth and development of children. Previous studies have suggested that spermidine supplementation may lead to varying degrees of improvement in weight status in high-fat diet-induced obese mice [17,18,20]. Moreover, an epidemiological study showed that spermidine intake was negatively associated with obesity [17]. Although serum spermidine and dietary spermidine are different, another study showed that the long-term oral intake of enhanced polyamine diets increases blood polyamine levels in both mice and humans [26]. Therefore, we hypothesize that serum spermidine and obesity are negatively correlated in humans.

However, the results of the cross-sectional study were contrary to the hypothesis. Compared with participants with lower serum spermidine concentrations, those with higher serum spermidine concentrations had a higher prevalence of obesity. We speculate that the elevated serum spermidine levels in the obese population are a compensatory effect in a pathological state. It has been suggested that oxidative stress is important in the pathophysiology of obesity and the increased oxidative stress contributes to the development and progression of obesity-related diseases [27,28]. Obesity is characterized by chronic low-grade inflammation. Spermidine has been shown to reduce oxidative stress damage by inhibiting inflammation and the accumulation of intracellular free radicals [29]. Our subgroup analysis also showed that the association between serum spermidine and obesity was only found in participants with obesity-related multimorbidity. Thus, the observed increase in serum spermidine levels in obese participants might act as a compensatory mechanism.

Our study confirmed the results reported by Codoñer-Franch et al. that polyamine levels are increased in childhood obesity and correlated to markers of oxidative/nitrosative stress and angiogenesis based on 102 children [30]. The previous study focused on childhood suggested that increased serum spermidine levels in obese children may be a compensatory effect, and our study suggested that this effect may also be present in adults. The different mechanism of compensation between children and adults needs to be explored by further studies in the future.

In the follow-up study, our findings are consistent with previous studies. The results of the follow-up study confirm the results of the above-mentioned study that spermidine has different degrees of protective effects against obesity. The protective effects of spermidine in obesity are closely related to autophagy and alleviate adipose tissue inflammation. Álvaro F Fernández et al. reported that pharmacological stimulation of autophagy by spermidine in mice reduced weight gain and obesity-related alterations under a high-calorie regimen [31]. Ma et al. found that spermidine reduced the inflammatory response of adipose tissue by decreasing the expression of inflammatory factors and chemokines [20]. Our findings suggest that participants with higher baseline serum spermidine concentrations are more likely to decrease BMI over two years, validating the protective effect of spermidine in obesity in the population.

In the follow-up study, we found a nonlinear relationship between spermidine and change in BMI. The trend after the peak point confirms the result of the cross-sectional study on the phenomenon of compensatory elevation of spermidine in obese patients. Whether the nonlinear relationship in the follow-up study correlates with the concentrations necessary for the metabolism and transport of spermidine still requires further study to determine the underlying mechanisms.

The main strengths of this study are as follows: (i) our results confirm previous experimental studies in a population; (ii) we used serum spermidine levels instead of spermidine estimated by the food frequency questionnaire to avoid self-reporting bias; (iii) we used a cross-sectional study and a follow-up study to increase the reliability of our results.

However, several limitations should be considered. First, we were unable to further adjust for the confounding effects of dietary spermidine and intestinal flora on the results, and these may affect the transportation and metabolism of spermidine. Second, serum spermidine was measured at a single time point, but disease onset and progression are complex and dynamic and may be accompanied by changes in spermidine. Third, we must acknowledge the preliminary nature of these results. We look forward to conducting further studies with longer follow-up periods and different populations in the future.

## 5. Conclusions

In conclusion, higher serum spermidine was associated with increased odds of obesity in the cross-sectional study but reduced odds for the increase in BMI in the follow-up study among Chinese adults. In the follow-up study, we found a reverse J-shaped relationship between spermidine and the change in BMI. Spermidine may be compensatorily elevated in obese people and have a protective effect against elevated BMI. In the future, more research is required to determine the exact mechanism underlying the association between spermidine and obesity and the scope for interventions. These results will aid in underlying the relationship between spermidine and obesity and will be of great utility in deciding whether to administer interventions.

## Figures and Tables

**Table 1:** Characteristics of participants at baseline by normal, overweight and obesity.

Characteristics	Normal (*n* = 1835)	Overweight (*n* = 1652)	Obesity (*n* = 743)	*p*-Value
Age, years	60.3 ± 10.1	58.8 ± 9.7	57.7 ± 0.7	<0.001
Female sex, *n* (%)	1120 (61.0%)	1075 (65.1%)	519 (69.9%)	<0.001
Ethnicity, *n* (%)				0.138
Han	1259 (68.6%)	1059 (64.1%)	452 (60.8%)	
Mongolian	502 (27.4%)	525 (31.8%)	260 (35.0)	
Others	74 (4.0%)	68 (4.1%)	31 (4.2%)	
Current smoking, *n* (%)	782 (42.6%)	574 (34.7%)	212 (28.5%)	<0.001
Current drinking, *n* (%)	417 (22.7%)	346 (20.9%)	148 (19.9%)	0.220
Physical labor levels, *n* (%)				0.841
Low	579 (31.6%)	549 (33.2%)	237 (31.9%)	
Moderate	1190 (64.9%)	1040 (63.0%)	478 (64.3%)	
High	66 (3.6%)	63 (3.8%)	28 (3.8%)	
Fruit/vegetables intake levels, *n* (%)				0.300
Few/day	92 (5.0%)	75 (4.5%)	35 (4.7%)	
250–500 g/day	898 (48.9%)	851 (51.5%)	371 (49.9%)	
500–1000 g/day	732 (39.9%)	634 (38.4%)	278 (37.4%)	
>1000 g/day	113 (6.2%)	92 (5.6%)	59 (7.9%)	
Whole grain intake levels, *n* (%)				0.141
Few/week	410 (22.3%)	306 (18.5%)	145 (19.5%)	
100–250 g/week	393 (21.4%)	370 (22.4%)	171 (23.0%)	
250–1000 g/week	458 (25.0%)	413 (25.0%)	191 (25.7%)	
>1000 g/week	574 (31.3%)	563 (34.1%)	236 (31.8%)	
Triglyceride, mmol/L	1.3 ± 1.1	1.8 ± 1.7	2.0 ± 1.9	<0.001
Total cholesterol, mmol/L	5.1 ± 0.9	5.2 ± 1.0	5.2 ± 1.0	<0.001
HDL-C, mmol/L	1.2 ± 0.3	1.1 ± 0.3	1.1 ± 0.2	<0.001
LDL-C, mmol/L	3.2 ± 0.8	3.3 ± 0.8	3.3 ± 0.8	0.035
Diabetes, *n* (%)	203 (11.1%)	264 (16.0%)	119 (16.0%)	<0.001
Stroke, *n* (%)	175 (9.5%)	222 (13.4%)	93 (12.5%)	0.001
Spermidine, ng/mL	23.8 (12.8–46.6)	25.3 (13.8–50.5)	27.2 (14.8–53.4)	0.002

HDL-C, high density lipoprotein cholesterol; LDL-C, low density lipoprotein cholesterol. Data are presented as mean ± SD, median (interquartile range) or *n* (%).

**Table 2:** OR and 95%CIs of serum spermidine levels for obesity (BMI ≥ 28 kg/m^2^) and overweight/obesity (BMI ≥ 24 kg/m^2^) in the cross-sectional study.

	Serum Spermidine						
	Q_1_	Q_2_		Q_3_		Q_4_	
	OR	OR (95% CI)	*p*-Value	OR (95% CI)	*p*-Value	OR (95% CI)	*p*-Value
Obesity							
Model 1	1.000 (Ref.)	1.009 (0.799, 1.275)	0.938	1.254 (1.001, 1.572)	0.049	1.307 (1.044, 1.636)	0.019
Model 2	1.000 (Ref.)	0.992 (0.785, 1.254)	0.946	1.263 (1.007, 1.584)	0.044	1.375 (1.096, 1.725)	0.006
Model 3	1.000 (Ref.)	0.993 (0.780, 1.264)	0.953	1.327 (1.050, 1.678)	0.018	1.417 (1.121, 1.791)	0.004
Overweight/Obesity							
Model 1	1.000 (Ref.)	1.096 (0.924, 1.301)	0.293	1.193 (1.005. 1.416)	0.044	1.312 (1.104, 1.559)	0.002
Model 2	1.000 (Ref.)	1.079 (0.909, 1.282)	0.385	1.200 (1.010, 1.426)	0.038	1.374 (1.154, 1.636)	<0.001
Model 3	1.000 (Ref.)	1.106 (0.919, 1.330)	0.288	1.315 (1.092, 1.582)	0.004	1.449 (1.201, 1.748)	<0.001

CI: indicates confidence interval; OR: odds ratio. Model 1: unadjusted. Model 2: adjusting gender and age. Model 3: adjusting gender, age, smoking, drinking, ethnicity, physical labor levels, fruit/vegetables intake levels, whole grain intake levels, triglyceride, HDL-C, LDL-C, total cholesterol, history of diabetes, history of stroke.

**Table 3:** OR and 95%CIs of baseline serum spermidine levels for the increase in body mass index in the follow-up study.

	Serum Spermidine						
	Q1	Q2		Q3		Q4	
	OR	OR (95% CI)	*p*-Value	OR (95% CI)	*p*-Value	OR (95% CI)	*p*-Value
Model 1	1.000 (Ref.)	0.956 (0.726, 1.259)	0.751	0.747 (0.567, 0.984)	0.038	0.505 (0.383, 0.666)	<0.001
Model 2	1.000 (Ref.)	0.933 (0.707, 1.230)	0.621	0.718 (0.544, 0.948)	0.019	0.494 (0.374, 0.653)	<0.001
Model 3	1.000 (Ref.)	0.931 (0.702, 1.235)	0.62	0.712 (0.535, 0.946)	0.019	0.493 (0.370, 0.657)	<0.001

CI: indicates confidence interval; OR: odds ratio. Model 1: adjusting baseline body mass index. Model 2: adjusting gender, age, baseline body mass index. Model 3: adjusting gender, age, baseline body mass index, smoking, drinking, ethnicity, physical labor levels, fruit/vegetables intake levels, whole grain intake levels, triglyceride, HDL-C, LDL-C, total cholesterol, history of diabetes, history of stroke.

## Data Availability

The data that support the findings of this study are available from the Northeast China Rural Cardiovascular Health Study (NCRCHS), but restrictions apply to the availability of these data, which were used under license for the current study, and so are not publicly available.

## Supplementary Materials

The following supporting information can be downloaded at: https://www.mdpi.com/article/10.3390/nu14132613/s1, Table S1: OR and 95%CIs of serum spermidine levels for overweight/obesity in the cross-sectional study; Table S2: OR and 95% CIs of baseline serum spermidine levels for the increase of body mass index by subgroups in the follow-up study.
