# Supplementary material for: Elevation of Serum Spermidine in Obese Patients: Results from a Cross-Sectional and Follow-Up Study
Source: Nutrients. 2022 Jun 24;14(13):2613. doi: 10.3390/nu14132613 (PMC9268142; doi:10.3390/nu14132613)
Supplement: Supplementary file 1 [file nutrients-14-02613-s001.zip › nutrients-1782056-supplementary.pdf]

Title: Elevation of Serum Spermidine in Obese Patients: Results from a Cross-Sectional and Follow-Up Study

Journal: Nutrients

Authors' Name:

Hanshu Gao[1,2#], MD; Qianlong Zhang[3#], MD; Jiahui Xu[2], MD; Wei Yuan[1], MD; Ruixue Li[1], MD; Hui Guo[1], MD; Cuiying Gu[1], MD; Wenjing Feng[1], MD; Yanan Ma[1,4], PhD; Zhaoqing Sun[5,*], PhD; Liqiang Zheng[2,*], PhD.

[#] Joint corresponding authors: Liqiang Zheng, Zhaoqing Sun

[#] Joint first authors: Hanshu Gao, Qianlong Zhang

Corresponding Author:

Liqiang Zheng, PhD.

School of Public Health, Shanghai Jiao Tong University School of Medicine, 227 Chongqing South Road, Huangpu District, Shanghai, 200025, China.

Tel.: +86-21-63846590

Fax: +86-21-63846590

E-mail: liqiangzheng@126.com

Zhaoqing Sun, PhD.

Department of Cardiology, Shengjing Hospital of China Medical University, Shenyang 110004, China;

Tel.: +86-24-83282688

Fax: +86-24-83282346

E-mail: sunzhaoqing@vip.163.com

The supplementary information is about some additional analyses of the main content of the article.

Table S1. OR and 95%CIs of serum spermidine levels for overweight/obesity in the cross-sectional study

	Serum spermidine						
	Q1	Q2		Q3		Q4	
	OR	OR (95%CI)	*P*-value	OR (95%CI)	*P*-value	OR (95%CI)	*P*-value
**Obesity**							
With multimorbidity	1.000 (Ref.)	1.081 (0.663, 1.763)	0.756	2.128 (1.332, 3.399)	0.002	2.143 (1.348, 3.405)	0.001
Without multimorbidity	1.000 (Ref.)	0.966 (0.729, 1.278)	0.807	1.131 (0.859, 1.489)	0.38	1.241 (0.941, 1.638)	0.126
**Overweight/obesity**							
With multimorbidity	1.000 (Ref.)	1.097 (0.740, 1.627)	0.643	1.736 (1.146, 2.631)	0.009	1.680 (1.121, 2.519)	0.012
Without multimorbidity	1.000 (Ref.)	1.116 (0.903, 1.379)	0.312	1.209 (0.980, 1.491)	0.077	1.373 (1.108, 1.701)	0.004

CI: indicates confidence interval; OR: odds ratio.

Models adjusting gender, age, smoking, drinking, ethnicity, physical labor levels, fruit/vegetables intake levels, whole grain intake levels, triglyceride, HDL-C, LDL-C, total cholesterol, history of diabetes, and history of stroke

Table S2. OR and 95% CIs of baseline serum spermidine levels for the increase of body mass index by subgroups in the follow-up study

	Serum spermidine						
	Q1	Q2		Q3		Q4	
	OR	OR (95%CI)	*P*-value	OR (95%CI)	*P*-value	OR (95%CI)	*P*-value
**Male**							
Model 1	1.000 (Ref.)	1.274 (0.755, 2.152)	0.365	1.010 (0.619, 1.649)	0.968	0.581 (0.365, 0.926)	0.022
Model 2	1.000 (Ref.)	1.253 (0.741, 2.120)	0.401	0.983 (0.600, 1.609)	0.944	0.587 (0.367, 0.937)	0.026
Model 3	1.000 (Ref.)	1.274 (0.736, 2.205)	0.388	0.942 (0.563, 1.576)	0.819	0.554 (0.339, 0.907)	0.019
**Female**							
Model 1	1.000 (Ref.)	0.857 (0.619, 1.186)	0.352	0.650 (0.466, 0.907)	0.011	0.475 (0.335, 0.673)	<0.001
Model 2	1.000 (Ref.)	0.831 (0.600, 1.151)	0.266	0.624 (0.446, 0.874)	0.006	0.464 (0.327, 0.658)	<0.001
Model 3	1.000 (Ref.)	0.818 (0.585, 1.143)	0.239	0.617 (0.436, 0.873)	0.006	0.476 (0.333, 0.682)	<0.001
**< 65 y**							
Model 1	1.000 (Ref.)	0.872 (0.622, 1.221)	0.424	0.736 (0.523, 1.036)	0.079	0.492 (0.349, 0.695)	<0.001
Model 2	1.000 (Ref.)	0.874 (0.624, 1.226)	0.436	0.714 (0.506, 1.006)	0.054	0.482 (0.341, 0.682)	<0.001
Model 3	1.000 (Ref.)	0.833 (0.588, 1.179)	0.302	0.693 (0.485, 0.989)	0.043	0.472 (0.329, 0.676)	<0.001
**≥ 65 y**							
Model 1	1.000 (Ref.)	1.057 (0.648, 1.724)	0.823	0.731 (0.455, 1.174)	0.194	0.514 (0.321, 0.824)	0.006
Model 2	1.000 (Ref.)	1.064 (0.652, 1.735)	0.804	0.734 (0.456, 1.180)	0.202	0.525 (0.326, 0.846)	0.008
Model 3	1.000 (Ref.)	1.141 (0.679, 1.917)	0.618	0.787 (0.475, 1.305)	0.354	0.505 (0.304, 0.839)	0.008
**Normal**							
Model 1	1.000 (Ref.)	0.836 (0.543, 1.287)	0.417	0.798 (0.516, 1.234)	0.311	0.586 (0.381, 0.901)	0.015
Model 2	1.000 (Ref.)	0.825 (0.535, 1.272)	0.384	0.777 (0.501, 1.206)	0.261	0.574 (0.372, 0.886)	0.012
Model 3	1.000 (Ref.)	0.763 (0.487, 1.196)	0.239	0.733 (0.464, 1.157)	0.182	0.563 (0.357, 0.887)	0.013

**Overweight**							
Model 1	1.000 (Ref.)	0.860 (0.567, 1.032)	0.475	0.655 (0.430, 0.997)	0.048	0.438 (0.287, 0.669)	<0.001
Model 2	1.000 (Ref.)	0.833 (0.548, 1.267)	0.393	0.613 (0.400, 0.938)	0.024	0.435 (0.284, 0.667)	<0.001
Model 3	1.000 (Ref.)	0.843 (0.546, 1.302)	0.44	0.644 (0.415, 0.999)	0.049	0.467 (0.298, 0.730)	0.001
**Obesity**							
Model 1	1.000 (Ref.)	1.768 (0.880, 3.536)	0.11	0.944 (0.471, 1.891)	0.871	0.506 (0.241, 1.062)	0.072
Model 2	1.000 (Ref.)	1.740 (0.860, 3.519)	0.123	0.960 (0.478, 1.931)	0.91	0.487 (0.230, 1.032)	0.06
Model 3	1.000 (Ref.)	1.825 (0.868, 3.837)	0.113	1.053 (0.495, 2.239)	0.893	0.470 (0.214, 1.034)	0.06

CI: indicates confidence interval; OR: odds ratio.

Model 1: adjusting baseline body mass index

Model 2: adjusting gender, age, and baseline body mass index

Model 3: adjusting gender, age, baseline body mass index, smoking, drinking, ethnicity, physical labor levels, fruit/vegetables intake levels, whole grain intake levels, triglyceride, HDL-C, LDL-C, total cholesterol, history of diabetes, and history of stroke
